# Prescribing antidepressants and anxiolytic medications to pregnant women: comparing perception of risk of foetal teratogenicity between Australian Obstetricians and Gynaecologists, Speciality Trainees and upskilled General Practitioners

**DOI:** 10.1186/s12884-020-03293-0

**Published:** 2020-10-14

**Authors:** Summer Williams, George Bruxner, Emma Ballard, Alka Kothari

**Affiliations:** 1 Gold Coast University Hospital, Southport, 4215 Queensland Australia; 2 Metro North Mental Health Service, Brisbane, 4006 Queensland Australia; 3 University of Queensland, St Lucia, 4067 Queensland Australia; 4 Queensland Institute of Medical Research, Berghofer Medical Research Institute, Brisbane, 4072 Queensland Australia; 5 Redcliffe Hospital, Anzac Avenue, Redcliffe, Queensland 4020 Australia

## Background

Depression and anxiety are common disorders, however their occurrence during pregnancy has the potential to significantly impact the health and wellbeing of both mother and child [1, 2]. Negative outcomes of mental health disorders in pregnancy include a variety of serious complications. Inadequately treated depression is associated with a substantial risk of maternal, fetal and neonatal morbidity and mortality [3]. In addition to subjective distress, the impact on relationships can be very significant, particularly when attachment to the newborn is disrupted. This may lead to enduring detrimental effects on the child extending into adulthood [4]. Depression also leads to suicide, with it being the second largest cause of indirect maternal mortality in the perinatal period in Australian women [1].

Unclear messages contribute to pregnant women being reluctant to take psychotropic medication, including antidepressants and anxiolytics with many fearing foetal harm [5–8]. Medical personnel including O&Gs and GPs form an important part of a pregnant woman’s network of information sources during pregnancy and can impact patient decision-making around medications in pregnancy [7–9]. The Australian clinicians’ own perception of teratogenicity of antidepressants (AD) and anxiolytics (AX) may influence counseling and care of vulnerable women and is largely unexplored. It is, however, likely to align with the international community where perceived teratogenicity is overestimated by physicians of all medical specialties, except psychiatrists [10–12]. Professional bodies such as the RANZCOG publish statements and recommendations to provide advice on management of perinatal anxiety and depression, serious mental illness and bipolar disorder. The target audience is all health professionals who are engaged in providing maternity and mental health care to these patients [13].

This study hypothesised that differences exist in the perception of risk of teratogenicity of AD and AX medication commonly prescribed to pregnant women, by differing clinicians, namely O&Gs and GPs. It also explored medication counselling and prescription practices, clinician resources and base knowledge of risk of AD and AX when used in pregnancy.

## Methods

### Setting and participants

Utilising the RANZCOG database, current Obstetrics and Gynaecology Fellows, trainees and “GP diplomates” (upskilled General Practitioners with additional qualifications in Women’s Health) were invited to participate in a nation-wide cross-sectional observational study of practices relating to prescription of AD and AX in pregnancy and provided a link to an anonymous ten-minute online questionnaire (www.surveymonkey.com) (Additional file 1). Participation was voluntary and consent was implied with completion and submission of the questionnaire. The responses submitted by the participants were de-identified. GP affiliates included in the study from New Zealand were virtually unrepresented, as they do not undertake the Diploma and were therefore not captured by this survey.

### Survey instrument

Our novel questionnaire was developed after researching questionnaire design and a directed literature search. Feedback was obtained from professional peers on the content and relevance of questions. A small pilot group of doctors (*n* = 10) tested the coherence of the questions, and the time frame to complete the questionnaire. The 34 questions were designed to elicit clinician attitudes about AD and AX including their prescription during pregnancy, medication counseling practice, perceptions of the level of patient concern regarding their use during pregnancy and the risk perceptions of the stakeholders who influenced a pregnant woman’s decision making. Demographic data was collected about the clinicians aligned specialty including their proportion of public and private practice, age, training, experience, interest in mental health and educational exposure. Clinician confidence in prescribing, managing adherence issues and perceived adequacy of training to manage depression and anxiety in pregnancy were also surveyed. Questions relating to attitudes and confidence were measured using Likert scales. Similar to published literature, we also included a series of questions to gauge basic AD and AX knowledge [2].

### Survey administration

The survey was adminstered through the RANZCOG and a reminder email was sent out 4 weeks after the initial invitation, reminding clinicians of the survey closure date.

### Statistical analysis

All data was analysed using the SPSS version 23 (IBM Corp., Armonk, NY). To aid with the interpretation of the questionnaire results, the following collapse of the Likert scale categories was made for Questions 21, 24 and 34: Agree = agree, strongly agree and Disagree = Strongly disagree, disagree and neutral. Categorical variables were summarised by frequency and percentage and continuous variables by mean and standard deviation (SD). Mean differences were reported with 95% confidence intervals (CI). Categorical variables were examined using Pearson Chi-squared test or Fisher’s exact test, where more than 20% of the expected values were less than 5. Continuous variables were checked for normality and examined using the Student t-test. Data was summarised for clinicians overall and separately by O&Gs and GPs. *P* values for the comparison of O&Gs and GPs were reported, with *p* < 0.05 considered to be statistically significant.

## Results

Overall, the RANZCOG database identified 5409 eligible clinicians, all of whom received a standardised invitation by email. This comprised of 2120 Fellows, 769 FRANZCOG trainees and 2520 Diplomates. A total of 545 valid responses were received and submitted for analysis (10.1%), less than the predicted response rate for medical personnel (32.8%) [12]. The response rate for O&G affiliates (12.9%) was consistent with gynaecologist rates from a similar risk perception study by Csajka et al. (13%) [2]. The response rate for GP affiliates was 6.8%.

### Demographics

Three hundred and seventy-three clinicians aligned with RANZCOG (68.4%) and 172 were affiliated with RACGP (31.6%). The demographic characteristics of the respondents are shown in Table 1. Seventy-two percent of respondents were trained in Australian medical colleges with 60.9% having over 10 years’ experience in their area of speciality. Twenty-six percent of O&Gs and 12.3% of GPs respondents had not yet attained their fellowship. Majority of the clinicians (98%) saw pregnant women in their clinical practice on a regular basis. Seventy-eight percent of O&Gs spent 11 h or more per week caring for pregnant women compared to 18.7% of GPs.

**Table 1:** Comparison of survey respondent demographics by clinical affiliation

Question	Overall	Obstetrician/ Gynaecologist	General Practitioner	***p***-value^
	n (%)	n (%)	n (%)	
	(*n* = 545)	(*n* = 373)	(*n* = 172)	
Age (years, *n* = 543)				0.011
23 to 30	47 (8.7%)	34 (9.2%)	13 (7.6%)	
31 to 40	182 (33.5%)	121 (32.6%)	61 (35.5%)	
41 to 50	116 (21.4%)	81 (21.8%)	35 (20.3%)	
51 to 60	129 (23.8%)	77 (20.8%)	52 (30.2%)	
61 or above	69 (12.7%)	58 (15.6%)	11 (6.4%)	
Years in specialty (including training) (*n* = 542)				0.61
< 11	212 (39.1%)	142 (38.4%)	70 (40.7%)	
11 or more	330 (60.9%)	228 (61.6%)	102 (59.3%)	
Where was medical student training completed? (*n* = 541)				< 0.001
Australia	391 (72.3%)	244 (65.9%)	147 (86.0%)	
New Zealand	46 (8.5%)	45 (12.2%)	1 (0.6%)	
Other	104 (19.2%)	81 (21.9%)	23 (13.5%)	
How long ago were the Fellowship training requirements completed? (*n* = 543)				0.003
Not yet completed	118 (21.7%)	97 (26.1%)	21 (12.3%)	
< 5	119 (21.98%)	79 (21.2%)	40 (23.4%)	
5 to 10	72 (13.3%)	43 (11.6%)	29 (17.0%)	
> 10	234 (43.1%)	153 (41.1%)	81 (47.4%)	
Working capacity? (*n* = 543)				< 0.001
Full time	407 (75.0%)	306 (82.5%)	101 (58.7%)	
Part time	132 (24.3%)	61 (16.4%)	71 (41.3%)	
No longer clinically active	4 (0.7%)	4 (1.1%)	0 (0.0%)	
You practice in a… (*n* = 543)				< 0.001
Public health facility	198 (36.5%)	182 (49.1%)	16 (9.3%)	
Private health facility	160 (29.5%)	72 (19.4%)	88 (51.2%)	
Both	185 (34.1%)	117 (31.5%)	68 (39.5%)	
Hours working with pregnant women per week (*n* = 541)				< 0.001
< 11	222 (41.0%)	83 (22.4%)	139 (81.3%)	
11 or more	319 (59.0%)	287 (77.6%)	32 (18.7%)	

^ *P*-value for comparison of Obstetrician/Gynaecologist versus General Practitioner

### Interest

In general, respondents had no particular interest in perinatal mental health disorders (36.7%), however more GPs (46.7%) were interested than O&Gs (32.1%). The vast majority of clinicians (96.9%) had not conducted any perinatal mental health research in the last 5 years. Also, fewer than half (46.4%) of all clinicians had attended a conference or read a journal article where AD or AX medication use in pregnancy had been reviewed. In general, only a small percentage of clinicians (15.3%) were involved in the provision of education to trainees about psychotropic prescription during pregnancy.

### Perception

Self-reported perception of concern around prescribing AD or AX medications was not significantly different between the groups (*p* = 0.38), with O&Gs (*n* = 368) apportioning a mean score of 3.7 (SD 2.3) and GPs (*n* = 169) a mean score of 3.9 (SD 2.4). This indicated a relatively low level of concern on a 0–10 scale, with 0 being no concerns. The perceived proportion of patient non-compliance was also not significantly different (*p* = 0.36) between the groups. Both of these estimated that just over a third of patients on a 0 to 100 scale would be non-compliant with their AD or AX treatment: O&Gs (*n* = 367) mean 34.8% (SD 18.7) and GPs (*n* = 170) 36.4% (SD 19.3). When asked to share their perceptions, GPs (*n* = 172) estimated their patients’ anxiety regarding AD and AX medication decision making in pregnancy as higher on a 0 to 100 scale: mean 73.7% (SD 21.3) compared with mean 63.1% (SD 24.1) for O&Gs (*n* = 372), a mean difference of 10.6% (95% CI 6.4–14.8).

### Practice

Only 10.5% of all clinicians (*n* = 545) “very often” provided pregnant women with written information about the intended prescription AD or AX. (6% of O&Gs compared to 14.5% of GPs). Sources of written information were varied and the overall numbers were small. Most of the O&Gs sourced UpToDate (32.2%), followed by MIMS (26.8%) and Mother Risk (13.4%). For GPs, the most commonly used resource was MIMS (27.9%) followed by “other” (19.2%) and Drug Company leaflets (15.1%). Less than 10% of all clinicians had their own practice pamphlets or relied on the pharmacists as their main source of written information. Thirty-two percent of O&Gs provided no written information compared with 16.3% of GPs (*p* < 0.001).

If seeing a pregnant patient with mental health illness for the first time, the time spent discussing potential maternal and foetal side effects of AD or AX treatment differed between clinician group (*p* < 0.001, *n* = 541). More than half of GPs (52.6%, *n* = 171) reported spending 15 min discussing potential maternal and foetal side effects of AD or AX treatment compared with O&Gs (48.6%, *n* = 370) spending less than 5 min.

There was a statistically and clinically significant difference (*p* < 0.001) in prescription practice where AD or AX initiation was surveyed: 84.8% of 171 GPs initiated these medications compared to 52.2% of 372 O&Gs.

The GPs ranked “prior response to the medicine” as being an influential reason (60.5%) for prescribing a particular AD or AX. O&Gs (*n* = 372) on the other hand, were more influenced by a medication “a mental health practitioner had previously prescribed” (50.5%). This preponderance for O&Gs to rank a specialist mental health clinicians’ opinion highly was also demonstrated later in the questionnaire, where 55.7% O&Gs (*n* = 357) would rely on the original prescriber’s management plan comapred to 11.7% of GPs (*n* = 162) (*p* < 0.001).

Responses to the question relating to discontinuation of fluoxetine in a hypothetical pregnant patient signified varying practices between clinician groups. Fifty-nine percent of GPs indicated that they would initiate a patient consultation compared to only 18.0% O&Gs. Furthermore, 48.8% of O&Gs suggested that they would seek referral to a mental health specialist compared to 5.3% of GPs.

### Confidence

**Fig. 1:**
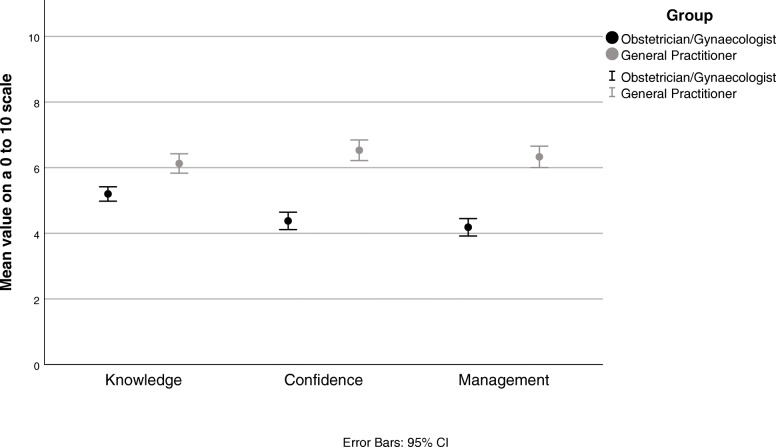
Comparison of self-reported knowledge and confidence in prescribing AD and AX medications by clinical affiliation

The questionnaire revealed that overall, clinicians’ main concerns regarding AD and AX medication prescription to women of reproductive age in order of perceived influence are: medical safety profile including teratogenicity (86.9%, *n* = 543), medical efficacy (75.2%, *n* = 537), neonatal adaption syndrome (70.0%, *n* = 543), and medication addiction potential (48.6%, *n* = 537). Of note, 57.4% of GPs (*n* = 169) were concerned about maternal side effects compared to 47.3% of O&Gs (*n* = 368) (*p* = 0.029) (Fig. 1).

There were differences in levels of reported confidence in being up-to-date with medication recommendations and safety profile with 57.6% of GPs feeling confident compared to 44.2% of O&Gs (*p* = 0.004). In general, GPs consider themselves to be more confident in their knowledge (mean difference 0.9 (95% CI 0.5–1.3) and ability to prescribe (mean difference 2.2 (95% CI 1.7–2.6) and manage (mean difference 2.1 (95% CI 1.7–2.6) AD and AX medications than O&Gs.

### Knowledge

Respondents were tested on their knowledge of five well-known AD and AX medications and their potential teratogenicity. As demonstrated in Table 2, GPs knowledge was generally similar to that of O&Gs, with the majority of respondents recognising that these medications had no significant proven teratogenicity. However, up to 22.3% respondents in both clinician groups incorrectly ascribed recognised teratogenicity to a commonly used AD or AX. Around 13% (*n* = 118) trainees were incorrect for sertraline, venlafaxine and diazepam while 28.2% (*n* = 117) were incorrect for amitriptyline and 21.2% (*n* = 118) for mirtazapine. Twelve percent of O&Gs considered “Sertraline” teratogenic compared to 3.5% of GPs (*p* = 0.001).

**Table 2:** Correct knowledge of teratogenicity of common AD and AX by clinical affiliation

Medication	Overall	Obstetrician/Gynaecologist	General Practitioner	***p***-value
	(*n* = 545)	(*n* = 373)	(*n* = 172)	
Sertraline (*n* = 542)	491 (90.6%)	325 (87.8%)	166 (96.5%)	0.001
Venlafaxine (*n* = 541)	471 (87.1%)	320 (86.7%)	151 (87.8%)	0.73
Amitriptyline (*n* = 537)	417 (77.7%)	286 (77.9%)	131 (77.1%)	0.82
Mirtazapine (*n* = 538)	444 (82.5%)	304 (82.8%)	140 (81.9%)	0.78
Diazepam (*n* = 542)	462 (85.2%)	313 (84.6%)	149 (86.6%)	0.53

### Training adequacy

GPs were more likely to agree that training and education had been adequate for them to feel confident in prescribing AD and AX to pregnant women (56.1%) compared to only a third of O&Gs (29.0%), *p* < 0.001. When asked what would be more useful to daily practice of caring for pregnant patients, 71.0% of all 541 respondents chose increased clinician education and training (71.1% O&Gs versus 70.8% GPs) in preference to increased technological supports such as apps for smart phones. Interestingly, 67.4% of a total of 543 clinicians agreed that completion of the study questionnaire had increased their interest in pursuing more information regarding AD and AX use in pregnancy.

## Discussion

Pregnant women with mental health conditions can be managed by a multitude of treatment modalities including psychosocial support and non-pharmacological interventions. This manuscript focusses on one aspect of the treatment- the use of psychotropic medications. To the best of our knowledge, this is the largest Australian survey of clinicians’ attitudes and practices, with regards to AD and AX prescription in pregnancy. It explores the differences between the two groups of medical practitioners, most frequently engaged in counseling pregnant women.

Appropriate management of anxiety and depression in pregnancy is an important area of clinical practice. If not properly addressed, it has the potential for deleterious irreversible consequences such as termination of pregnancy and maternal suicide [1]. Over 50% of pregnancies are unintended and may be associated with an increased risk of postpartum depression [14].. Untreated anxiety and depression during pregnancy is associated with increased weight gain, substance abuse and smoking [15]. Pregnant women with antenatal anxiety and depression are less likely to attend regular antenatal appointments and have higher complications such as stillbirth, premature birth, low birth weight and low Apgar scores [3, 15, 16]. Engagement of pregnant women with perinatal mental health services remains an everlasting challenge with the added concern of patient initiated sudden cessation of medication [17]. Hence, clinician’s confidence and competence in adequately treating anxiety and depression in pregnancy is very important.

Considerable uncertainty in prescribing AD and AX in pregnancy exists, even amongst clinicians with expertise in antenatal health care provision [2, 9, 11]. Women in general also express extreme reluctance to take medication in pregnancy [7, 8, 17]. Both clinician groups in this study felt that training had not been adequate to instil confidence in medication prescription, even though many health professionals had trained for more than 10 years. Both groups advocated for improved training to address this need.

This study suggests there may be differences in perception, confidence and practice between clinician groups. GPs perceived higher rates of patient anxiety regarding AD and AX use in pregnancy, and felt that they had a greater influence on a women’s use of AD or AX in pregnancy. Even though they saw pregnant women less frequently, they reported that their consultations apportioned more time to discussing medication risk. GPs less frequently expressed an intent to refer to a mental health specialist, most likely reflecting their role as primary prescribers. They also ranked the influence of their psychiatric colleagues lower than O&Gs and the impact of the internet. GPs reported higher rates of confidence in managing mental health conditions in pregnant patients at a community-level compared to their O&G counterparts, perhaps due to their familiarity with medication manipulation and more frequent provision of mental health advice for general patients [12].

Both groups, in practice, recommended close doctor-patient relationships to nurture clear communication and support during the pregnancy, and no groups ill-advisedly recommended ceasing AD or AX upon pregnancy or for lactation. Both groups perceived women’s fears about foetal malformation when AD or AX use in pregnancy was raised. However, it is concerning that 9.4 to 22.3% of clinicians incorrectly labelled commonly used AD or AX medication as causing teratogenicity. This highlights the need for ready access to updated, evidence-based sources of medication advice for clinicians.

Provision of written information has a strong evidence base supporting benefits for patient decision making, especially in a population group where anxiety or lack of concentration may cause impairment [13]. Our study shows that this resource is infrequently used (~ 10%). There was no universal patient and clinician-friendly source from where the information was obtained. This likely reflects the difficulty of finding robust evidence regarding medication use in pregnancy, which is likely a consequence of ethical restraints on trialling medications in pregnant women [18]. The onus remains on the clinicians to update themselves with latest available data.

The participants who responded to the survey admitted only a modest interest in mental health disorders in pregnancy. They also admitted to not being actively involved in research, nor had their knowledge of treatments challenged often by new data at conferences or in journal articles. In addition, they were infrequently involved in passing on that knowledge to trainees. This lack of familiarity may have led to both clinical affiliates overestimating perceived teratogenicity of commonly used psychotropic medications.

## Limitations

Due to the low response rates and inherent limitations such as including self-selected groups of respondents in such surveys, the findings of this study should be interpreted with caution. The number of responders is however not trivial and their perceptions around the prescription of AX an AD during pregnancy clearly suggests a need for further research in this very important area of medicine. The authors also acknowledge that grouping broad groups of antidepressants and anxiolytics is also a potential limitation of the study, however the very high level of comorbidity of anxiety and depressive symptoms and the anxiolytic properties of antidepressants made a general focus on these medication groups a practical and less potentially confusing approach,

## Conclusion

In pregnancies complicated by mental health conditions requiring AD or AX treatment, GPs are potentially more confident discussing and prescribing these medications compared to their O&G counterparts. Nevertheless, with nearly a quarter of clinicians overestimating the teratogenicity of a commonly used AD, training could be improved for both GPs and O&G affiliates. This would assist with optimal management of anxiety and depression in pregnancy for the benefit of the mother and unborn child.

## Supplementary information

**Additional file 1.**

## Data Availability

The questionnaire generated and analysed during the current study is not publicly available due to the HREC not providing consent but is available from the corresponding author on reasonable request.

## Abbreviations

AD: Antidepressants
AX: Anxiolytics
O&G: Obstetrician and Gynaecologist
GP: General Practitioner
RANZCOG: Royal Australian College of Obstetricians and Gynaecologists
