# Supplementary material for: Prescribing antidepressants and anxiolytic medications to pregnant women: comparing perception of risk of foetal teratogenicity between Australian Obstetricians and Gynaecologists, Speciality Trainees and upskilled General Practitioners
Source: BMC Pregnancy Childbirth. 2020 Oct 14;20:618. doi: 10.1186/s12884-020-03293-0 (PMC7556911; doi:10.1186/s12884-020-03293-0)
Supplement: Supplementary file 1 — Additional file 1. [file 12884_2020_3293_MOESM1_ESM.pdf]

Prescribing antidepressant and anxiolytic medications to pregnant women: the perception of risk of fetal teratogenicity amongst Australian specialists and trainees

Thank you for your participation in this online survey. It should take less than 10 minutes to complete. Your answers are confidential and may inform an understanding of Australian and New Zealand practice.

Please answer every question.

1. What college are you currently affiliated with?

- ◯ RANZCOG
- ◯ RACGP
- ◯ ACRRM
- ◯ Other (please specify)

2. Do you see pregnant patients in your clinical practice?

- ◯ yes
- ◯ no

3. What is your age?

- ◯ 23 - 30
- ◯ 31 - 40
- ◯ 41 - 50
- ◯ 51 - 60
- ◯ 61 or above

4. How many years have you been working in your area of specialty? (include your training years)

- ◯ less than 5 years
- ◯ 5 - 10 years
- ◯ 11 - 20 years
- ◯ > 20 years

5. Where did you complete your medical student training?

- ○ New Zealand
- ○ Australia
- ○ Other (please specify)

6. When did you complete your final Fellowship training requirements?

- ○ I have not completed them yet
- ○ Less than 5 years ago
- ○ 5 - 10 years ago
- ○ Greater than 10 years ago

7. In the last 12 months, in what capacity were you predominantly working?

- ○ full time role
- ○ part time role
- ○ no longer clinically active
  (if this is the case, please answer the subsequent questions with reference to your prior typical practice)

8. You practice in a...

- ○ public health facility
- ○ private health facility
- ○ both

9. On estimate, how many hours of your working week involves caring for pregnant women?

- ○ less than 1 hour
- ○ < 5 hours
- ○ 5 - 10 hours
- ○ 11 - 25 hours
- ○ >25 hours

10. Do you have any particular interest in mental health disorders in pregnancy?

- ○ yes
- ○ no

11. Have you conducted any research into perinatal mental health disorders in the past 5 years?

- ◯ yes
- ◯ no

12. In the last 6 months have you attended a conference where prescribing antidepressant or anxiolytic medication in pregnancy was discussed or have you read a journal article reviewing this topic?

- ◯ yes
- ◯ no

13. Are you involved in the provision of education to trainees regarding antidepressant or anxiolytic medication in pregnancy, in the form of tutorials, lectures or presentations?

- ◯ yes
- ◯ no

14. Do you provide pregnant patients with written information about the antidepressant and anxiolytic medication you prescribe to them?

- ◯ never
- ◯ rarely
- ◯ sometimes
- ◯ very often

15. What are your sources of the written information?

- ☐ pamplets made by your practice or department
- ☐ drug company information leaflets
- ☐ UpToDate patient information
- ☐ MIMS database
- ☐ The MotherRisk website
- ☐ you rely on the pharmacist to supply this
- ☐ you don't provide written information

Other (please specify)

____

16. Do you feel up to date with the latest medication recommendations and safety profiles?

- ◯ yes
- ◯ no

17. Using the scale below, indicate what percentage of pregnant women you perceive are anxious about prescribed antidepressant and anxiolytic medication decision-making in pregnancy.
(Drag the slider to the preferred position, or enter a numerical rating in the textbox.)

0% 50% 100%

18. For women already on prescribed antidepressant or anxiolytic medications during their pregnancy, indicate the proportion you believe are not compliant with their treatment.
(Drag the slider to the preferred position, or enter a numerical rating in the textbox.)

0% 50% 100%

19. If seeing a pregnant patient with mental illness for the first time, how long do you spend discussing potential antidepressant and anxiolytic medication treatment side effects (maternal and fetal)?

- ◯ less than 5 minutes
- ◯ around 15 minutes
- ◯ most of the consultation

20. On a scale of 0 to 10, where 0 equals "no concerns" and 10 equals "extremely concerned", how would you rank your level of concern about prescribing antidepressant or anxiolytic medication to pregnant patients and the risk of fetal harm?

0 5 10

21. "This medicine has recognised teratogenicity". Please respond to this statement for each of the medications below.

	strongly disagree	disagree	neutral	agree	strongly agree
sertraline (Zoloft®)	◯	◯	◯	◯	◯
venlafaxine (Efexor®)	◯	◯	◯	◯	◯
amitriptyline (Endep®)	◯	◯	◯	◯	◯
mirtazapine (Avanza®)	◯	◯	◯	◯	◯
diazepam (Valium®)	◯	◯	◯	◯	◯

22. Have you ever initiated an antidepressant or anxiolytic medication for a pregnant patient?

◯ yes

◯ no

23. What are the main factors that would influence your decision to prescribe a particular antidepressant or anxiolytic medication to a pregnant patient? Choose three

☐ known safety profile of medication

☐ experience in prescribing that medication

☐ patient preference

☐ the gestational age of the pregnancy

☐ prior response to this medication

☐ cost

☐ efficacy

☐ known teratogenicity profile

☐ interactions with other medications

☐ a mental health practitioner has previously prescribed this medication

24. Please respond to this statement.
"Your main concerns regarding antidepressant and anxiolytic medication prescription to women of reproductive age are..."

	strongly disagree	disagree	neutral	agree	strongly agree
the maternal side effects	○	○	○	○	○
the medications' efficacy	○	○	○	○	○
the medications' safety profile including fetal teratogenicity	○	○	○	○	○
the medications addiction potential	○	○	○	○	○
neonatal adaption syndrome or other neonatal withdrawal syndrome	○	○	○	○	○

25. Indicate yes or no.
"If prescribing antidepressant or anxiolytic medication to a pregnant woman, you usually..."

	yes	no
cease it upon diagnosis of pregnancy	○	○
continue the same dose throughout	○	○
increase dosing in 2nd or 3rd trimester as dictated by symptoms	○	○
taper the medication before the womens' due date	○	○
cease the medication for lactation	○	○
rely on the original prescribers' management plan	○	○

Other

26. If a patient asks to reduce her dose or cease an antidepressant or anxiolytic medication during pregnancy, regardless of her stated reason for doing this, what is your perception of the most influential motivations for this action? Choose one.

- ○ medication was unnecessary
- ○ medication use might result in neonatal withdrawal
- ○ previous experience in previous pregnancy
- ○ friend or family pressure
- ○ fear about incidence of fetal malformations
- ○ fear about impact on breastfeeding
- ○ Other (please specify)

27. In your experience, who or what seems to be the most influential on a womens opinion of her use of antidepressant and anxiolytic medication? Choose three.

- ☐ her own rationale
- ☐ television
- ☐ partner, friend or family member
- ☐ her GP
- ☐ an internet web page
- ☐ magazine articles
- ☐ her specialist psychiatrist or mental health practitioner
- ☐ her work colleagues
- ☐ her obstetrician or midwife
- ☐ written information from the hospital

Other (please specify)

28. If your patient wishes to discontinue their fluoxetine during the pregnancy, you would....

- ◯ arrange a consultation with yourself
- ◯ educate the patient and advise to continue the same dose
- ◯ reduce the dose to strike a balance between maternal concerns and the underlying need for treatment
- ◯ recommend discontinuation of the medication
- ◯ consider change to another antidepressant
- ◯ refer to their mental health practitioner to address this concern
- ◯ you don't manage pregnant patients

29. For the next 3 questions, respond to the statement, indicating your level of confidence, where 0 equals no confidence and 10 equals a high level of confidence.

"You have a good base knowledge about the safety profile and efficacy of antidepressant and anxiolytic medication

0 5 10

30. "You feel confident prescribing antidepressants to pregnant women"

0 5 10

31. "You confidently manage pregnant patients with anxiety and prescribe medications when you feel necessary"

0 5 10

32. Do you feel the training and education you have undertaken so far has been adequate to confidently prescribe antidepressant and anxiolytic medications to pregnant patients?

- ◯ yes
- ◯ no

33. Which do you think may be more useful to your daily practice of caring for pregnant patients? Choose one.

- ◯ greater development of technological aids such as iPhone or android apps with advice about medication and fetal risks for pregnant women
- ◯ improved education and training for clinicians so they can anticipate issues and counsel pregnant patients well

34. Upon completion of this survey, you have increased your interest in furthering your knowledge of prescribing to pregnant patients with mental health concerns

	strongly disagree	disagree	neutral	agree	strongly agree
You....	◯	◯	◯	◯	◯

You've now completed our survey. Thank you for your participation! It is greatly appreciated.

Dr Summer Williams
Dr George Bruxner
Dr Alka Kothari
